# Membrane transporters modulating the toxicity of arsenic, cadmium, and mercury in human cells

**DOI:** 10.26508/lsa.202402866

**Published:** 2024-11-22

**Authors:** Andrè Ferdigg, Ann-Katrin Hopp, Gernot Wolf, Giulio Superti-Furga

**Affiliations:** 1 CeMM Research Center for Molecular Medicine of the Austrian Academy of Sciences, Vienna, Austria; 2 Center for Physiology and Pharmacology, Medical University of Vienna, Vienna, Austria

## Abstract

Non-essential metals are extremely toxic to living organisms, posing significant health risks, particularly in developing nations where they are a major contributor to illness and death. Although their toxicity is widely acknowledged, the mechanisms by which they are regulated within human cells remain incompletely understood. Specifically, the role of membrane transporters in mediating heavy metal toxicity is not well comprehended. Our study demonstrates how specific transporters can modulate the toxicity of cadmium, mercury, and the metalloid arsenic in human cells. Using CRISPR/Cas9 loss-of-function screens, we found that the multidrug resistance protein MRP1/ABCC1 provided protection against toxicity induced by arsenic and mercury. In addition, we found that SLC39A14 and SLC30A1 increased cellular sensitivity to cadmium. Using a reporter cell line to monitor cellular metal accumulation and performing a cDNA gain-of-function screen, we were able to clarify the function of SLC30A1 in controlling cadmium toxicity through the modulation of intracellular zinc levels. This transporter-wide approach provides new insights into the complex roles of membrane transporters in influencing the toxicity of arsenic, cadmium, and mercury in human cell lines.

## Abstract (precis)

This study employs CRISPR/Cas9 loss-of-function screens to simultaneously investigate the contribution of the entire transportome to regulating the toxicity of arsenic, cadmium, and mercury in human cell lines.

## Introduction

Heavy metals are a key component of most, probably all, biological systems (Rossetto & Mansy, 2022). Their chemical property and versatility has led to their participation in a vast spectrum of cellular processes, including cellular respiration, oxygen metabolism, metabolic pathways, and protein biosynthesis (Jomova et al, 2022). However, several of the heavy metals most abundant in nature lack a known biological role. These non-essential metals, among them, the species cadmium, mercury, and the metalloid arsenic, are highly toxic, and they represent an underestimated threat to public health, accounting for millions of deaths every year (Jarup, 2003). The primary source of chronic exposure to toxic metals for humans remains the consumption of contaminated food and water (Bridges & Zalups, 2005). For example, arsenic, recognized as one of the most prevalent chemical contaminants in drinking water, has been linked to increased rates of infant mortality and cancer (Zheng, 2020). Likewise, mercury poses a growing concern in child development, particularly through the ingestion of contaminated fish (Bose-O’Reilly et al, 2010). Furthermore, cadmium is believed to contribute to the development of lung cancer and cardiovascular diseases because of the consumption of tobacco (Kim et al, 2023).

Non-essential metals exert promiscuous effects on cells at a molecular level. The main common feature is an increase in oxidative stress through several mechanisms that unbalance the cellular redox state (Valko et al, 2005). For example, they deplete cells of glutathione through direct chelation and through inhibition of several antioxidant enzymes, such as superoxide dismutase and glutathione reductase (Chrestensen et al, 2000; Rubino, 2015; Bovio et al, 2021). The ability of non-essential metals to inhibit the respiratory chain, leading to the accumulation of unstable semiquinones and hence the formation of superoxides, is well documented (Belyaeva et al, 2012). A screen in yeast scoring for protein aggregates by imaging has identified several components of the proteostatic machinery as effector targets (Andersson et al, 2021). Finally, non-essential metals have been found to interfere with the homeostasis of essential trace elements by competing for their binding site on metalloproteins (Jan et al, 2015). Interestingly, metals can also act as stimulators of signaling pathways, such as the pathways involving the MAPK/Erk kinases, contributing to the expression of proto-oncogenes and facilitating cellular transformation (Matsuoka & Igisu, 2002).

Substantial candidate-driven research has allowed the identification of key molecular targets and modulators of metal toxicity. These include not only metal chelators such as metallothioneins and metal chaperones, but also antioxidant enzymes, metabolic enzymes, and membrane transporters (Günther et al, 2012; Yu et al, 2017; Douillet et al, 2023). The importance of membrane transporters in maintaining cellular metal homeostasis is well described in the literature (Nelson, 1999). Furthermore, mutations in heavy metal transporters are known to have systemic effects in humans, leading to severe phenotypes differentially affecting specific organs (Petrukhin et al, 1993; Tuschl et al, 2016).

Most studies on the effect of non-essential metals, including their traffic through membrane transporters, have focused on individual proteins and candidates so that despite a plethora of evidence and information, unbiased studies or studies evaluating whole classes of proteins in parallel are rare. Consequently, it is reasonable to hypothesize that many molecular regulators of metal toxicity may have not yet been identified and that the individual contribution of any such regulator to the overall protective effect remains relatively unfathomed. Investigating the pleiotropic effects of metal toxicity requires unbiased methodologies capable of simultaneously assessing a multitude of genes.

The few genetic screening approaches that have been applied to study metal-induced toxicity over the last decade have mainly employed model organisms, such as yeast or silkworm (Du et al, 2015; Sobh et al, 2019; Andersson et al, 2021; Liu et al, 2021). As a consequence, although several transporters for non-essential metals are annotated in the literature (Himeno et al, 2009; Bridges & Zalups, 2010), the function of membrane transporters in mediating metal toxicity in human cells has not been addressed systematically yet.

Here, we apply CRISPR/Cas9 loss-of-function (LOF) genetic screens to investigate the involvement of membrane transporters in regulating the toxicity of arsenic, mercury, and cadmium in human cell lines. Through this approach, we uncover the genetic dependency of human cell lines on individual membrane transporters when experiencing toxic stress by non-essential metals. Our findings emphasize the importance of transporters in maintaining cellular metal homeostasis and mitigating the toxic effects of non-essential metals.

## Results

### Toxicity of arsenic, cadmium, and mercury in human cancer cell lines

**Figure 1.**
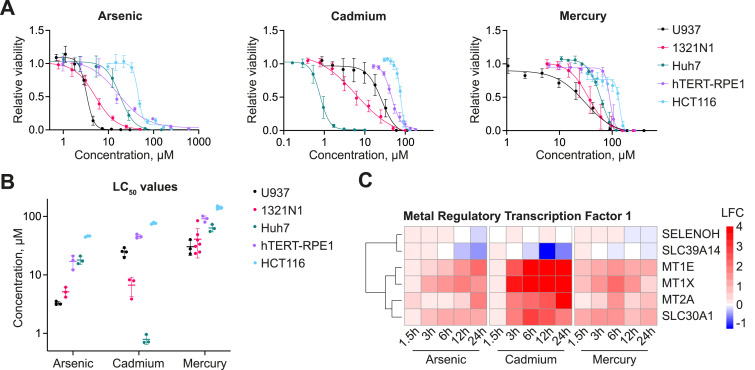
Toxicity characterization and transcriptional response to arsenic, cadmium, and mercury. **(A)** Viability curves of human cell lines exposed to a gradient of arsenic, cadmium, or mercury for 48 h. **(B)** LC_50_ values derived from the sigmoidal fitting curves in (A). Each dot represents an independent replicate. **(C)** Heatmap showing the differential expression of the literature-curated gene set of the metal regulatory transcription factor 1 filtered for genes expressed in the 1321N1 cell line upon treatment with arsenic, cadmium, or mercury for the annotated amount of time. Data are the mean ± SD from at least three independent experiments.

To gain a first insight into the toxicity profile of the non-essential metals cadmium, mercury, and the metalloid arsenic (hereinafter collectively referred to as metals), we decided to investigate their impact on the viability of a panel of human cancer and immortalized cell lines. We selected cell lines originating from different organs known to be targets of metal poisoning, namely, Huh7 (liver, hepatocellular carcinoma), 1321N1 (brain, astrocytoma), U937 (blood, histiocytic lymphoma), HCT116 (colon, carcinoma), and the hTERT-immortalized cell line RPE1 (eye, retinal pigment epithelial cells). We performed a viability assay upon treatment with a concentration gradient of arsenic, cadmium, and mercury for a duration of 48 h. All three metals elicited a sigmoidal viability curve in the human cell lines tested (Fig 1A). Furthermore, by comparing the corresponding LC_50_ values we observed a strong variability of toxicity (Fig 1B). First, the same cell line showed a different degree of sensitivity to each metal, with arsenic having a slightly higher toxicity and mercury showing the lowest degree of toxicity. A similar toxicity profile had been reported previously for human cell lines (Egiebor et al, 2013; Karri et al, 2018), hinting at the existence of metal-intrinsic features that dictate the toxicity profile, such as possible differences in the mechanisms of toxicity. Secondly, different cell lines manifested different sensitivity for the same metal (Fig 1A and B). For example, the high sensitivity of the liver cell line Huh7 to cadmium was striking (Fig 1A, mid-panel). In contrast, the colon carcinoma cell line HCT116 exhibited the lowest sensitivity to all three metals among all cell lines (Fig 1A and B). Taken together, the varying sensitivity of the five cell lines and the difference in toxicity between the three metals suggested that (i) there may be cell type–specific differences contributing to metal toxicity, and/or (ii) toxicity may be mediated by metal-intrinsic features, including differences in the mechanisms of toxicity and the respective cellular responses.

### Arsenic, mercury, and cadmium induce a similar transcriptional profile

**Figure S1.**
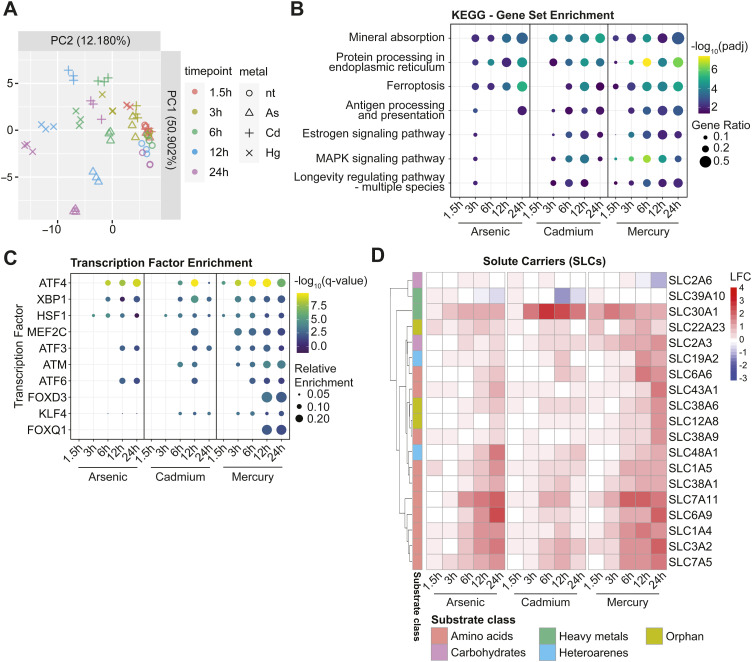
Transcriptional analysis of cellular response to heavy metal treatment. **(A)** PCA plot showing the individual replicates of the transcriptional analysis of 1321N1 cells treated with arsenic, cadmium, or mercury for designated time. **(B)** Gene set enrichment analysis using the Kyoto Encyclopedia of Genes and Genomes ordered by decreasing normalized enrichment score of the 1321N1 cell line treated with arsenic, cadmium, or mercury for the annotated amount of time. Disease-related terms were omitted. **(C)** Bubble plot displaying the enrichment analysis of the transcription factor target genes for the transcriptome dataset from (A), as determined by the TRRUST (v2) database. **(D)** Heatmap showing the differential expression of solute carriers with a LFC > 1 or < −1 upon treatment with arsenic, cadmium, or mercury for the annotated time. Substrate class annotation based on Goldmann et al (2024) *Preprint*.

If different metals affect cells differently, they may have non-identical cellular targets, and this should become manifest in the overall transcriptional response. We asked the question whether the three metals could be distinguished by the patterns and kinetics of the transcriptional profiles they elicit. Given that astrocytes are a crucial target of metal toxicity (Li et al, 2021a), we opted to focus on the astrocyte-derived cell line 1321N1. Although transcriptional profile analysis is a common approach for studying metal-induced responses in human cell lines and higher organisms (Ung et al, 2010; Gasser et al, 2022), many studies often focus on single time points of treatment, neglecting the temporal dimension and, consequently, the kinetics of toxicity (Singh et al, 2021). Here, we performed time-resolved 3′ mRNA sequencing on the 1321N1 cell line exposed to equitoxic concentrations (here, LC_20_) of arsenic, cadmium, or mercury. To get a first overview of the overall differences between metals and treatment times, we performed principal component analysis on the transcriptome dataset, which revealed distinct time- and metal-dependent transcriptional changes (Fig S1A). We then analyzed the canonical response to heavy metals, focusing on genes known to be controlled by the metal regulatory transcription factor 1 (MTF1), a pivotal coordinator of heavy metal response. MTF1 mitigates metal toxicity through the expression of the metal-chelating metallothioneins, metal membrane transporters, and antioxidant selenoproteins (Günther et al, 2012). We monitored the transcriptional changes of the literature-curated genes regulated by MTF1, filtering for those genes that were expressed in the 1321N1 cell line. We observed a robust up-regulation of several metallothioneins (*MT1E*, *MT1X*, *MT2A*) as early as 3 h from the start of the treatment, along with an increase in the zinc exporter *SLC30A1* (also known as *ZNT1*; Fig 1C). In addition, we observed a minor down-regulation of the zinc importer *SLC39A10* (*ZIP10*), consistent with MTF1’s function in repressing its expression. MTF1 functions as an intracellular zinc sensor, and its activation by non-essential metals is mediated, among other factors, by the release of zinc from intracellular stores induced by the non-essential metals competing for zinc-binding sites (Zhang et al, 2003). In line with this, we noted that cadmium was the most potent activator of MTF1, presumably because of its very high affinity to the zinc-binding sites of metallothioneins (Waalkes et al, 1984).

To further investigate potential differences in affected pathways between metals and time points, we performed gene set enrichment analysis (GSEA) using the KEGG gene set (Ogata et al, 1999; Subramanian et al, 2005). Significant pathway enrichment was evident as early as 3 h after treatment start, or as early as 1.5 h in the case of mercury (Fig S1B). Across all three metal responses, the term associated with *protein processing in endoplasmic reticulum* was consistently observed among the top enriched terms. This aligned with our transcription factor enrichment analysis, where we determined the relative enrichment of target genes using the TRRUST dataset (Han et al, 2018). In this analysis, we noted that the two primarily enriched transcription factors were ATF4 and XBP1, both linked to endoplasmic reticulum stress and the unfolded protein response (Fig S1C). In line with the proposed mechanism of metal toxicity via oxidative stress, all three metals showed enrichment of the term linked to ferroptotic cell death (Fig S1B). Non-essential metals are known activators of the estrogen and MAPK signaling pathways. We indeed observed activation of both pathways by cadmium and mercury, but not arsenic. The most prominently enriched term was associated with uptake and distribution of minerals, highlighting the importance of metal membrane transporters and metal-binding proteins in the cellular responses to non-essential metals. This prompted us to analyze the differential regulation of membrane transporters upon metal treatment in more detail. Indeed, we observed up-regulation of several solute carrier (SLC) membrane transporters (Fig S1D), implying a potentially crucial role of membrane transporters in adapting to metal-induced toxicity. Intriguingly, only two out of the 20 differentially regulated SLCs were annotated as metal transporters, with the majority being amino acid transporters. Cadmium was the most potent inducer of the zinc exporter *SLC30A1*. Conversely, mercury and arsenic stimulated the expression of amino acid transporters required for the biogenesis of glutathione (Fig S1D, bottom cluster), among them, the heterodimers *SLC7A5/SLC3A2* and *SLC7A11/SLC3A2*, as well as *SLC6A9*, and *SLC1A4*. Overall, arsenic, cadmium, and mercury elicited a comparable transcriptional response, with similar kinetics. Consequently, the observed difference in toxicity (Fig 1B) should more likely be attributable to cell type–intrinsic factors, such as differential gene expression, splicing, and genetic variation, rather than differences in the molecular responses of toxicity.

### Transporter-focused loss-of-function screen identifies many known players

**Figure 2.**
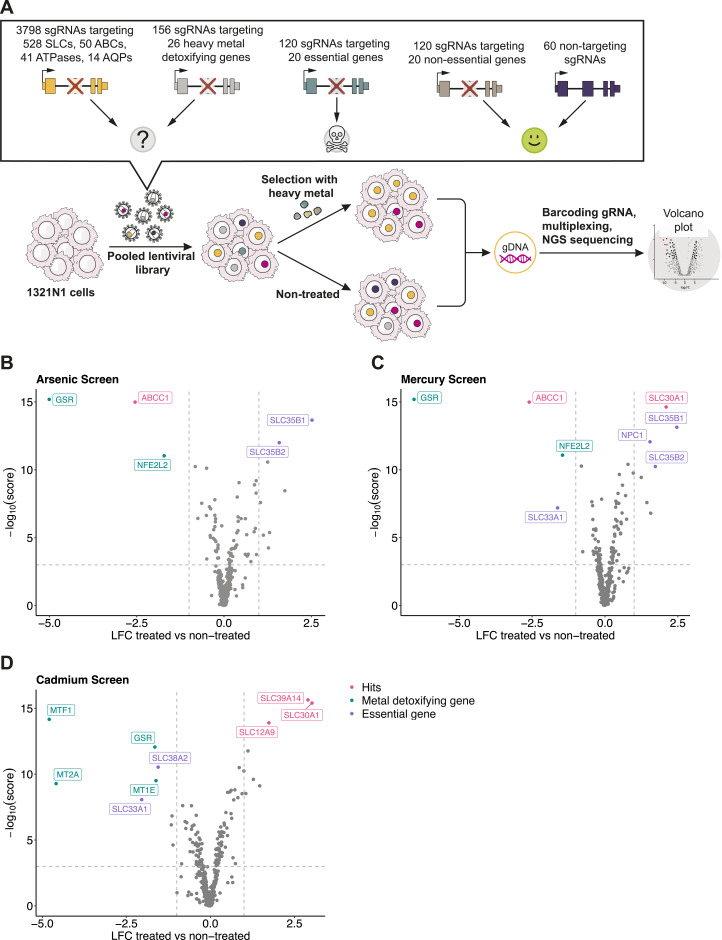
Membrane transporter–focused CRISPR/Cas9-KO screens identify transporters regulating the toxicity of arsenic, mercury, and cadmium. **(A)** Experimental setup of the heavy metal CRISPR/Cas9 survival screens in the 1321N1 cell line. **(B, C, D)** Volcano plots showing the enrichment or depletion of all sgRNAs per gene as log_2_ fold change (LFC) and the MAGeCK robust rank aggregation score (−log_10_ score) in cells exposed to toxic concentrations of annotated heavy metals relative to non-treated cells.

After having shown that arsenic, cadmium, and mercury induced overall similar transcriptional responses, we asked whether they rely on distinct genetic factors to mediate their cellular toxicity. We decided to focus on membrane transporters, considering their pivotal role in modulating metal homeostasis (Kambe et al, 2008; Pizzagalli et al, 2021), the term “mineral absorption” enriched in our GSEA of the response transcriptome (Fig S1B), and the observed up-regulation of several SLC transporters upon treatment (Fig S1D). We conducted transporter-focused CRISPR/Cas9 screens to investigate the genetic dependency of the human cell line 1321N1 in the context of metal toxicity. For our screens, we employed a sgRNA library targeting membrane transporter superfamilies known to regulate metal transport in human cell lines. This is a larger collection than the one we previously used in similar studies (Sedlyarov et al, 2018; Girardiet al, 2020b; Li et al, 2021b), to include, beyond SLCs, also ATP-binding cassette (ABC) transporters, P-type ATPases, and aquaporins (AQPs) (Wolf et al, manuscript submitted). We included genes involved in the detoxification of metals as positive controls (Table S1). Upon lentiviral delivery of the pooled library, transduced 1321N1 cells were exposed to toxic concentrations of arsenic, cadmium, or mercury for 6 d. Changes in the sgRNA abundance relative to the non-treated cells were assessed by deep sequencing (Fig 2A).

Table S1. List of the transporter-focused CRISPR/Cas9 sgRNA library including a sgRNA sublibrary targeting known heavy metal control genes.

**Figure S2.**
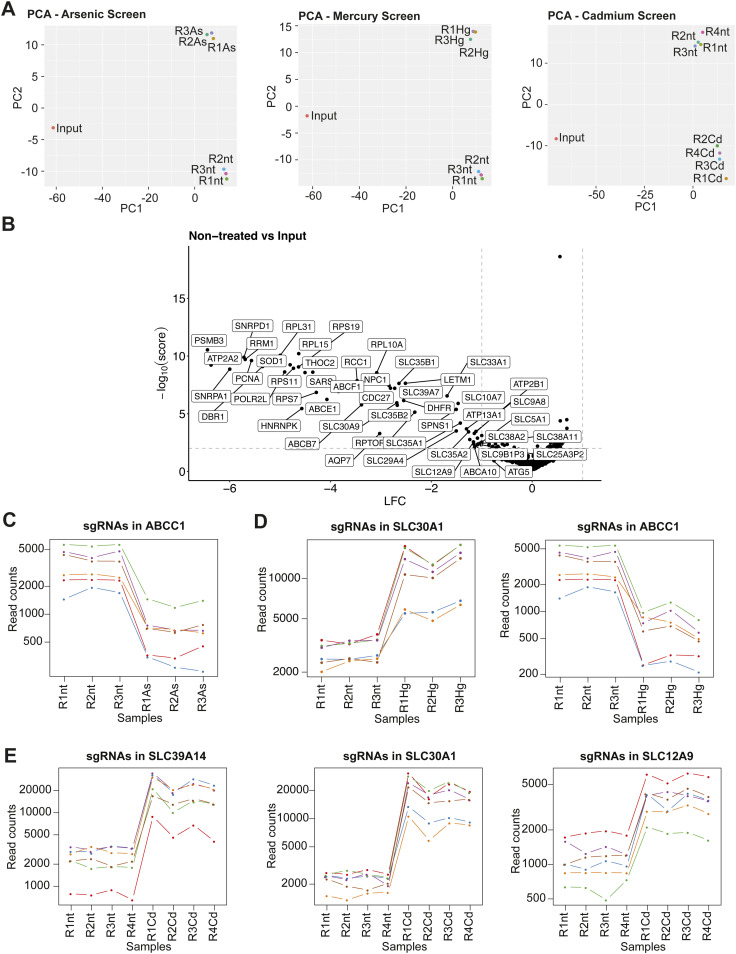
Quality control of the membrane transporter–focused genetic screens of cells exposed to heavy metals. **(A)** PCA plots of the sgRNA counts from the CRISPR/Cas9 screens showing the individual replicates of metal-treated and non-treated conditions, as well as the plasmid library stock (Input). **(B)** Volcano plots showing the median enrichment of all sgRNAs per gene as log_2_ fold change (LFC) and the MAGeCK robust rank aggregation score (−log_10_ score) in non-treated conditions relative to the plasmid library stock (Input). **(C, D, E)** Read counts of individual sgRNAs across replicates from the CRISPR/Cas9 screens of the hits chosen for arrayed validation of both non-treated and arsenic (C)-, mercury (D)-, or cadmium (E)-treated conditions.

To gain an overview of the overall difference in sgRNA abundance, we performed principal component analysis, which revealed reproducible segregation of the treated replicates from the input plasmid library stock and the non-treated cells, assuring the overall quality and reproducibility of the experiments (Fig S2A, Table S2). Comparing the sgRNA distribution in the non-treated cells with the library plasmid stock showed that several sgRNAs targeting essential control genes and essential membrane transporters led to a growth reduction (Fig S2B), confirming a high KO efficiency in this cell line and experimental setup. To identify genes that may modulate the toxicity of arsenic, cadmium, or mercury, we calculated the differential sgRNA abundance in treated compared with non-treated cells. As expected, we observed a depletion of several sgRNAs targeting the control genes, specifically the metal-detoxifying genes (Fig 2B–D). Glutathione reductase (*GSR*) was depleted in all three screens, albeit to different extents, genetically underscoring the importance of glutathione in mitigating metal toxicity. The cadmium exposure genetic screen showed depletion of sgRNAs targeting the transcription factor *MTF1* and its downstream targets, the metallothioneins *MT2A* and *MT1E* (Fig 2D). This further validates the experimental setup and genetically ratifies the role of metallothioneins in dampening cadmium toxicity (Klaassen et al, 2009). In line with this, we observed a strong activation of MTF1 by cadmium in our transcriptomic analysis (Fig 1C).

Table S2. MAGeCK read counts of the CRISPR/Cas9 loss-of-function screens.

Treatment with the non-essential metals resulted in both enrichment and depletion of cells lacking membrane transporters, as suggested by the enrichment or depletion of the respective sgRNAs, implying that some transporters were increasing, and others decreasing, the toxicity of the three metals (Fig 2B–D, Table 1). Several of these membrane transporter hits are shared among cells treated with arsenic or mercury. The sgRNAs targeting the multidrug resistance protein 1 (*ABCC1*, *MRP1*) were depleted upon treatment with both metals (Figs 2B and C and S2C and D), in line with the role of ABCC1 in mediating the efflux of arsenic and mercury conjugated to glutathione (Leslie et al, 2004; Straka et al, 2016). Furthermore, both metals led to the enrichment of sgRNAs targeting intracellular, essential membrane transporters, particularly members of the SLC35 family. The proteins SLC35B1 and SLC35B2 are involved in regulation of ER homeostasis and protein glycosylation/sulfation through ATP/ADP exchange and nucleotide-sugar transport in the ER and Golgi (Kamiyama et al, 2003; Klein et al, 2018). Inactivation of these genes, considered to be essential for cellular fitness, is likely to have pleiotropic effects, and therefore, the link to arsenic/mercury toxicity might be attributed to several proteins downstream of SLC35B1/B2, for example, to proteins depending on SLC35B1/B2 activity for folding or localization. In addition, mercury-treated cells showed a minor enrichment of cells lacking the zinc exporter *SLC30A1*. On the contrary, cadmium treatment led to the enrichment of sgRNAs targeting the cadmium transporter *SLC39A14*, the zinc exporter *SLC30A1*, and, to a lesser extent, the potassium chloride transporter *SLC12A9* (Figs 2D and S2E). We further observed depletion of the two essential transporters, *SLC38A2* and *SLC33A1*. Considering that these genes are essential, as evidenced by comparing the sgRNA abundance in non-treated cells with the library plasmid stock (Fig S2B), the observed phenotype is most likely unrelated to metal toxicity. Overall, our CRISPR/Cas9 screens provide a global assessment of the genetic dependency of a human cell line on membrane transporters when exposed to arsenic, mercury, or cadmium, highlighting the potential roles of individual transporters in mediating or mitigating toxicity.

**Table 1.** Overview of the genes scoring in the CRISPR/Cas9 loss-of-function screens.

Heavy metal	Hit	Substrate	Subcellular localization	Essential gene
Arsenic	ABCC1	GSH conjugates	PM	No
	SLC35B1	UDP-glucuronic acid	ER	Yes
	SLC35B2	Phospho-adenylyl sulfate	Golgi	Yes
Mercury	ABCC1	GSH conjugates	PM	No
	SLC30A1	Zinc	PM	No
	SLC35B1	UDP-glucuronic acid	ER	Yes
	SLC35B2	Phospho-adenylyl sulfate	Golgi	Yes
	NPC1	Cholesterol	Golgi, lysosome, endosome	Yes
	SLC33A1	Acetyl-CoA	ER	Yes
Cadmium	SLC39A14	Manganese, zinc, cadmium, iron	PM, endosome	No
	SLC30A1	Zinc	PM	No
	SLC12A9	Potassium chloride	PM, intracellular	No
	SLC38A2	Amino acids, sodium	PM	Yes
	SLC33A1	Acetyl-CoA	ER	Yes

All non-essential genes (marked in pink) were selected for arrayed validation. Substrate annotation based on Goldmann et al (2024) *Preprint*. PM, plasma membrane; ER, endoplasmic reticulum.

### Validation of transporters in regulating metal sensitivity

**Figure 3.**
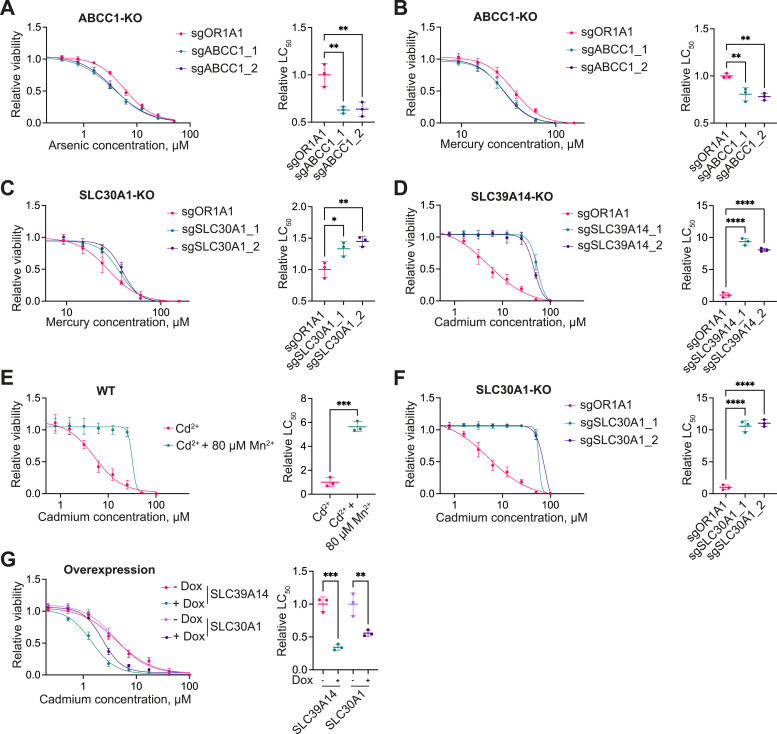
Validation of membrane transporter hits. **(A, B, C, D, F)** Viability curves normalized to non-treated cells and corresponding LC_50_ values derived from the sigmoidal fitting curve of 1321N1 KO cell pools expressing two different sgRNAs targeting the gene of interest or the control sgRNA targeting the olfactory receptor *OR1A1*. KO cell pools were exposed to arsenic (A), mercury (B, C), or cadmium (D, F) for 48 h. **(E)** Viability curves of the 1321N1 parental cell line co-treated with cadmium and 80 μM manganese(II) chloride for 48 h. Data were normalized on the cadmium–non-treated condition. **(G)** Viability curves and corresponding LC_50_ values of 1321N1 cells overexpressing indicated cDNAs in a doxycycline (Dox)-inducible fashion that were seeded in the presence or the absence of doxycycline and treated with cadmium for 48 h. Data are the mean ± SD from three independent experiments. ns *P* > 0.05, *P ≤ 0.05, **P ≤ 0.01, ***P ≤ 0.001, ****P ≤ 0.0001.

**Figure S3.**
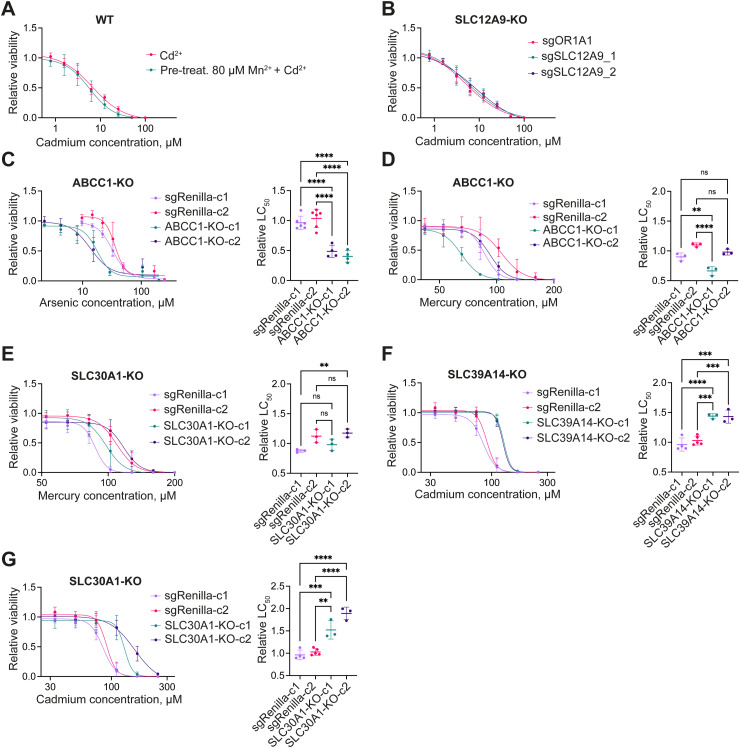
Further validation of membrane transporter hits. **(A)** Viability curves of the 1321N1 parental cell line pre-treated with 80 μM manganese(II) chloride for 24 h, followed by 48 h of cadmium treatment. Data were normalized on the cadmium–non-treated condition. **(B)** Viability curves of the 1321N1 KO cell pools expressing two different sgRNAs targeting the gene of interest or the control sgRNA targeting the olfactory receptor *OR1A1*. The KO cell pools were exposed to cadmium for 48 h. **(C, D, E, F, G)** Viability curves and corresponding LC_50_ values of the HCT116 KO clones and clones bearing the non-cutting control sgRNA Renilla. The clones were treated for 48 h with arsenic (C), mercury (D, E), or cadmium (F, G). Data were normalized on the non-treated condition and are shown as the mean ± SD from three independent experiments. ns *P* > 0.05, *P ≤ 0.05, **P ≤ 0.01, ***P ≤ 0.001, ****P ≤ 0.0001.

We selected all non-essential membrane transporter hits from the primary screens for arrayed validation (Table 1). The validation strategy involved generation of KO 1321N1 cell pools using the two highest scoring sgRNAs for each gene (Fig S2C–E), followed by viability measurements upon treatment with arsenic, mercury, or cadmium. All KO pools were confirmed by TIDE-coupled Sanger sequencing. KO of the efflux pump *ABCC1* significantly sensitized cells to arsenic and mercury compared with the control cell line bearing the sgRNA targeting the unrelated gene olfactory receptor 1A1 (*OR1A1*) (Fig 3A and B). Furthermore, cells depleted of the zinc exporter *SLC30A1* showed a significant growth advantage when exposed to toxic concentrations of mercury (Fig 3C), validating the enrichment of this mutant observed in the pooled CRISPR screen. As for cadmium-treated cells, we observed a roughly ninefold shift in the LC_50_ value for cells lacking *SLC39A14*, compared with the control *OR1A1*-KO cell line (Fig 3D). In addition to its ability to transport cadmium, SLC39A14 is a known importer of essential trace elements, among them, iron, zinc, and manganese (Tuschl et al, 2016). It was previously reported that manganese may protect from cadmium toxicity in cultured cells and reduce cellular cadmium accumulation, presumably because of competition for uptake via SLC39A14 (Himeno et al, 2009). Indeed, we observed that co-treatment of 1321N1 WT cells with manganese efficaciously rescued cadmium toxicity to a similar extent as cells lacking *SLC39A14* (Fig 3E). Pre-treatment with manganese did not confer protection from cadmium toxicity (Fig S3A), excluding the possibility that the growth rescue upon co-treatment was solely mediated by indirect effects, such as transcriptional adaptation to manganese. Surprisingly, inactivation of the zinc exporter *SLC30A1* gene resulted in a comparable growth advantage upon exposure to cadmium (Fig 3F). In line with this, we observed sensitization to cadmium upon the cDNA overexpression of *SLC39A14* and *SLC30A1* (Fig 3G). Finally, KO of *SLC12A9* had no effect on cell growth behavior upon exposure to cadmium (Fig S3B).

To investigate whether the observed effects of individual transporters on metal toxicity were cell type–specific, we conducted the same validation experiments in an additional cell line. For convenience, we decided to use HCT116 KO clones from the RESOLUTE consortium, as they were readily available and had been amply validated and characterized (Superti-Furga et al, 2020). We selected two different KO clones per target gene and compared their sensitivity with two clones carrying a non-targeting Renilla sgRNA. Results for arsenic-treated cells were successfully reproduced, showing a sensitizing effect upon *ABCC1*-KO (Fig S3C). In addition, we observed a growth rescue in cadmium-treated cells bearing inactivation of the *SLC39A14* or *SLC30A1* genes (Fig S3F and G). However, the variation between the two cell clones was too high to observe a consistent effect of *ABCC1*-KO and *SLC30A1*-KO on the viability of mercury-treated cells (Fig S3D and E). In summary, all non-essential membrane transporter hits, except for *SLC12A9*, were successfully validated in the 1321N1 cell line, suggesting that the observed phenotype may have general validity. Hits from the arsenic and cadmium screens could be reproduced in the HCT116 cell line, whereas the minor protective effect of *SLC30A1*- and *ABCC1*-KO on mercury toxicity observed in the 1321N1 cell line was potentially masked by the clonal variation in the HCT116 KO clones.

### Role of SLC30A1 in cadmium toxicity

**Figure 4.**
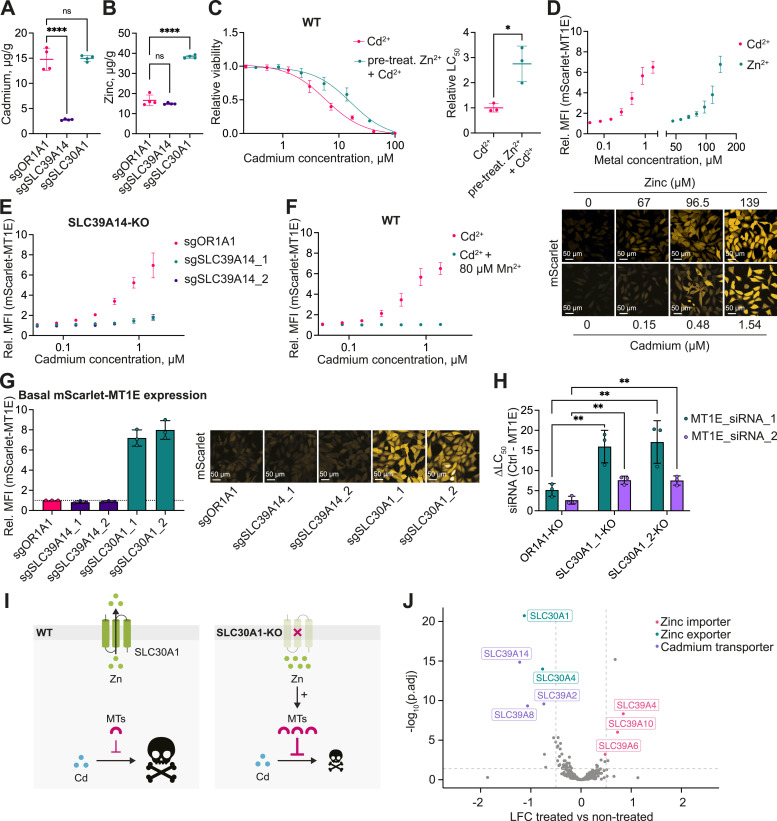
Metal exporter SLC30A1 regulates cadmium toxicity through modulation of intracellular zinc levels. **(A)** Total cellular cadmium levels measured by ICP-MS in 1321N1 cells expressing sgRNAs targeting *SLC39A14* and *SLC30A1*, or the control sgRNA targeting *OR1A1* treated with 1 μM cadmium chloride. Data were normalized on cell pellet weight and are represented as the mean ± SD from four replicates harvested on the same day. **(B)** Total cellular zinc levels measured by ICP-MS in untreated cells from (A). **(C)** Viability curve and LC_50_ values derived from the corresponding sigmoidal fitting curve of 1321N1 parental cells treated with cadmium for 48 h or pre-treated with 150 μM zinc chloride for 24 h, followed by 48 h of cadmium treatment. **(D)** Mean fluorescence intensity (MFI) and representative pictures of endogenously tagged mScarlet-metallothionein 1E (*mScarlet-MT1E*) 1321N1 cells treated with cadmium or zinc for 24 h relative to non-treated cells. **(E)** MFI of mScarlet-MT1E cells expressing either one of two sgRNAs targeting *SLC39A14* or the control sgRNA targeting the olfactory receptor *OR1A1* treated with cadmium for 24 h. **(F)** MFI of mScarlet-MT1E cells treated with cadmium or co-treated with 80 μM manganese(II) chloride for 24 h relative to the non-treated condition. **(G)** MFI of untreated mScarlet-MT1E cells bearing sgRNAs targeting *SLC30A1* or *SLC39A14* relative to a control cell line expressing sgOR1A1, and corresponding representative pictures. **(H)** Difference in LC_50_ between control siRNA and siRNA targeting *MT1E* in 1321N1 cell lines expressing sgOR1A1 or sgRNAs targeting *SLC30A1* derived from the sigmoidal fitting curves in Fig S4E. **(I)** Model of cadmium protection via zinc-mediated induction of metallothioneins (MTs) in *SLC30A1*-KO cells. **(J)** Volcano plot of the cDNA-based overexpression screen in the 1321N1 cell line exposed to toxic concentrations of cadmium showing the enrichment of all cDNAs per gene as log_2_ fold change (LFC) and the significance (−log_10_ *P* adj) relative to non-treated cells. Data are the mean ± SD from at least three independent experiments. *P ≤ 0.05, **P ≤ 0.01, ****P ≤ 0.0001.

**Figure S4.**
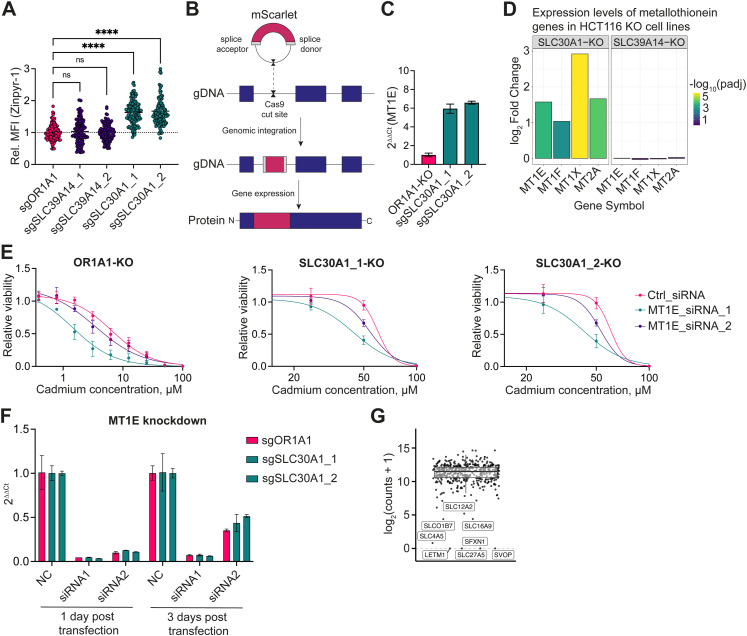
Metallothionein expression levels change in response to zinc. **(A)** Mean fluorescence intensity of the zinc-specific sensor Zinpyr-1 in 1321N1 cells expressing sgRNAs targeting *SLC30A1* or *SLC39A14*, relative to cells expressing sgRNA targeting the control gene *OR1A1*. Each dot represents an individual cell. **(B)** Scheme depicting the generation of the mScarlet-MT1E 1321N1 cell line by endogenous tagging of the first intron of the metallothionein 1E gene locus with mScarlet. **(C)** Expression levels of *MT1E* in negative control siRNA-transfected cells from the qRT-PCR (F) normalized on sgOR1A1-expressing cells. Data are represented as the mean ± SD of two technical replicates. **(D)** Expression levels of different metallothionein genes based on the publicly available transcriptomic dataset of the RESOLUTE consortium of HCT116 *SLC30A1*- and *SLC39A14*-KO clones. **(E)** Viability curves of 1321N1 cells bearing one of two sgRNAs targeting *SLC30A1* or the control *OR1A1* upon siRNA-mediated knockdown of *MT1E* treated with cadmium for 48 h. **(F)** qRT-PCR analysis showing the knockdown efficiency of metallothionein *MT1E* in 1321N1 *SLC30A1*-KO and *OR1A1*-KO cells, as assessed by the 2^ΔΔCt^ method. The expression levels were normalized on the non-targeting, negative control (NC) siRNA relative to the reference gene *HPRT1* and are represented as the mean ± SD of two technical replicates. **(G)** Distribution of the SLC-focused cDNA library in the transduced 1321N1 cell pool before the start of the heavy metal treatment (Table S4, R1–4_Day0). Labeled cDNAs possessed low counts and were excluded from the analysis.

Intrigued by the observation that the KO of the annotated zinc exporter *SLC30A1* strongly rescued cells from cadmium toxicity, we decided to further dissect the underlying protective mechanism. Zinc has previously been reported to protect against cadmium-induced toxicity (Limaye & Shaikh, 1999; Zhang et al, 2014; Yu et al, 2020). Hence, we reasoned that SLC30A1 could be mitigating cadmium toxicity through the modulation of cellular zinc levels. Alternatively, in addition to its ability to transport zinc, the transporter might also be able to regulate cellular cadmium levels via direct transport. To address this latter point, we measured total cellular zinc and cadmium levels using inductively coupled plasma mass spectrometry (ICP-MS) in *OR1A1*-, *SLC30A1*-, and *SLC39A14*-KO cells treated with 1 μM cadmium. The ionomics data revealed a significant reduction in cellular cadmium levels in *SLC39A14*-KO cells compared with the control cell line, whereas the loss of *SLC30A1* did not affect cadmium levels (Fig 4A). Regarding zinc levels, untreated *SLC30A1*-KO cells exhibited ∼2.3-fold higher total cellular zinc compared with the control cell line, whereas zinc levels remained unchanged in *SLC39A14*-KO cells (Fig 4B). Similarly, we detected an increase in cellular zinc levels in live *SLC30A1*-KO cells using the zinc-specific fluorescent sensor Zinpyr-1 (Fig S4A), consistent with previous reports (Moskovskich et al, 2019). These findings suggest that SLC30A1 is not involved in cadmium uptake, and its protective role against cadmium toxicity is likely because of indirect effects.

We then asked the question whether the observed viability rescue of *SLC30A1*-KO cells upon exposure to cadmium could be mediated by the increased intracellular zinc levels. Indeed, pre-treatment of WT 1321N1 cells with zinc protected the cells from cadmium toxicity (Fig 4C). These protective effects may be mediated by metallothioneins, which are up-regulated in response to elevated cellular levels of zinc and cadmium (Zhang et al, 2014; Yu et al, 2020), through the activation of the transcription factor MTF1 (Smirnova et al, 2000; Zhang et al, 2003; Günther et al, 2012). Based on the transcriptional profile of cadmium-treated cells, we observed a strong up-regulation of several metallothioneins (Fig 1C) and hypothesized that zinc accumulation would induce the same metallothionein genes. To assess metallothionein protein abundance in response to *SLC30A1* depletion, we selected metallothionein 1E (*MT1E*) as a reporter gene and inserted an artificial exon encoding for mScarlet within the first intron of the *MT1E* gene in the 1321N1 cell line (Fig S4B). Upon treatment of the reporter cell line with zinc, we observed an increase in fluorescence (Fig 4D), a phenotype also seen upon cadmium treatment. Notably, inactivation of the metal importer gene *SLC39A14*, responsible for cadmium uptake, completely prevented cadmium-induced metallothionein expression (Fig 4E), in line with the ionomics results (Fig 4A). A similar effect was observed in WT cells when cadmium uptake was competed with manganese (Fig 4F). Depleting the reporter cell line of *SLC30A1* resulted in a roughly sevenfold increase in basal mScarlet-MT1E expression in non-treated cells (Fig 4G), presumably because of the increased intracellular zinc levels. The control cell line *OR1A1*-KO, as well as the *SLC39A14*-KO cell line, failed to lead to this increase. We confirmed the up-regulation of untagged *MT1E* in 1321N1 *SLC30A1*-KO cells by quantitative RT-PCR (qRT-PCR) (Fig S4C). In addition, the publicly available RESOLUTE RNA-sequencing dataset (https://re-solute.eu/resources/datasets) also revealed the up-regulation of several metallothioneins, including *MT1E*, in HCT116 *SLC30A1*-KO but not *SLC39A14*-KO cells (Fig S4D).

This supported the notion that the observed phenotype in *SLC30A1*-KO cells was mediated by effects related to intracellular accumulation of zinc, such as the up-regulation of metallothioneins. If this was indeed the case, then inactivation of metallothionein genes should sensitize cells to cadmium. Knockdown of *MT1E* indeed led to a stronger sensitization to cadmium in *SLC30A1*-KO cells compared with the *OR1A1*-KO cell line, as indicated by the change in LC_50_ (Figs 4H and S4E). The smaller effect of siRNA#2 could be attributed to the lower knockdown efficiency toward the end of the cadmium treatment period, as shown by qRT-PCR (Fig S4F). On the contrary, knockdown of *MT1E* only partially sensitized *SLC30A1*-KO cells to cadmium. This could be explained by the presence of other metallothionein proteins, as well as metallothionein-independent protective effects, such as the antioxidant properties of zinc (Souza et al, 2004). Taken together, the zinc exporter SLC30A1 modulated cadmium toxicity by regulating intracellular zinc levels, which in turn induced protective effects, such as the up-regulation of metallothioneins (Fig 4I).

If indeed zinc was the main protector against cadmium-induced toxicity in the *SLC30A1*-KO cells, then modulation of intracellular zinc levels via genetic alteration of the expression levels of other zinc transporters should lead to a comparable phenotype. Yet, no other zinc transporters scored in the CRISPR LOF screen (Fig 2D), presumably because of the pronounced redundancy of zinc transporters, as well as their low expression levels in the 1321N1 cell line. To circumvent this impediment, we performed a SLC-focused, pooled overexpression screen based on a doxycycline-inducible cDNA library (Figs 4J and S4G; Table S3). This SLC-wide approach should assess whether any potential cadmium transporter that did not score in the LOF screen would be revealed by this positive functional mode. Upon delivery of the library to the 1321N1 cell line, cells were treated with a toxic concentration of cadmium in the presence of doxycycline and the distribution of the cDNAs was determined after 5 d of treatment (Table S4). Cells overexpressing *SLC30A1* and *SLC39A14* showed the strongest depletion from the cell pool (Fig 4J). This gain-of-function experiment using a different technology (cDNA expression versus LOF) strongly validated the role of *SLC30A1* and *SLC39A14*. Furthermore, we identified two additional cadmium transporters, *SLC39A2* and *SLC39A8*, which did not score in the CRISPR screen. Regarding the modulation of intracellular zinc levels, cells overexpressing zinc importers, such as *SLC39A4*, *SLC39A6*, and *SLC39A10*, were significantly enriched, whereas the overexpression of the zinc exporters *SLC30A4* and *SLC30A1* led to depletion from the cell pool. Hence, membrane transporters that regulate cellular zinc levels determined the sensitivity to cadmium toxicity, underscoring the importance of the biological cross-regulation of these metals.

Table S3. cDNA and barcode sequences of the SLC overexpression library.

Table S4. MAGeCK read counts of the SLC cDNA-based overexpression screen.

## Discussion

Heavy metals are a double-edged sword: they are essential for human life, and yet can be toxic already at relatively low concentrations (Rossetto & Mansy, 2022). Especially non-essential metals, which do not have a known biological function, among them cadmium, mercury, and the metalloid arsenic, are highly toxic to biological systems (Bridges & Zalups, 2005). Here, we systematically investigated the toxicity of these three metals in human cell lines, with a specific focus on the potential role of membrane transporters in modulating toxicity. Our findings revealed varying degrees of toxicity among the metals, as well as differences in sensitivity across different cell types. To which extent the toxicity is driven by metal-intrinsic properties, differential genetic predisposition or by the gene expression profile of the different cell types is difficult to address. Our transcriptomic analysis revealed that all three metals induced a comparable transcriptional response with similar kinetics. In addition, to pinpoint the membrane transporters involved in modulating arsenic, cadmium, and mercury toxicity, we conducted focused LOF screens. Through this genetic approach that considers 633 transporters in parallel (including 79 pseudogenes), we were able to functionally assess the involvement of known metal transporters and uncovered indirect mechanisms that help cells cope with heavy metal toxicity.

We conducted a comparative, time-resolved transcriptomic analysis of a human cell line treated with the three metals. Although this may admittedly represent a poor proxy for the toxicity mechanisms, it is reflecting a broad biological response that can be conveniently compared in a quantitative and temporal way. In our study, it showed that the three metals displayed comparable kinetics, allowing to speculate that the kinetics of induced toxicity are similar. The overall transcriptional response was comparable across the different metals. This aligns with recent transcriptomic studies, which have shown that the primary affected pathways are largely independent of the specific heavy metal species or tissue origin (Fatema et al, 2021; Forcella et al, 2022). Could the transcriptomic analysis provide some insights into the differences in toxicity by the different metals? Lower enriched terms often differ between the cell lines and metals (Fatema et al, 2021; Forcella et al, 2022). These differences could be ascribed to many causes. The cell lines used originate from different individuals, so there could be variants contributing to the observed differences. Furthermore, the different cell identity and differentiation state will have an influence on the metal responses. Together, this points to the fact that the genetic makeup, as well as the differences in secondary mechanisms of toxicity between the metals, which are potentially masked by promiscuous effects like oxidative stress, defines the toxicity profile. Future studies should also consider differences in protein translation efficiency and protein homeostasis across different cell types and metals (Tamás et al, 2014), as metal-induced protein aggregation has been suggested as a mode of action for promoting neurodegenerative diseases (Breydo & Uversky, 2011; Savelieff et al, 2013). Future investigations will undoubtedly also shed more light on the biochemical nature of metal-induced cell death, an aspect that we did not investigate.

In our CRISPR screens, we successfully identified known heavy metal transporters, including the metal importer SLC39A14, which mediates the uptake of cadmium in addition to manganese, iron, and zinc (Girijashanker et al, 2008), and the multidrug resistance protein MRP1/ABCC1, responsible for the export of mercury–glutathione and arsenic–glutathione conjugates (Leslie et al, 2004; Granitzer et al, 2020). This underlines the importance of membrane transporters as regulators of cellular metal homeostasis. Nevertheless, some of the transporters for cadmium, mercury, and arsenic annotated in the literature did not score significantly in our screen. On top, the strongest hits were not membrane transporter, but soluble proteins (GSR and MTF1). Both observations can largely be attributed to the substrate redundancy of transporters (Pizzagalli et al, 2021), which compensates for the absence of individual transporters, thereby preventing significant depletion or enrichment in a LOF genetic screen. In addition, the outcomes of LOF screens are inherently influenced by the expression profile of the used cell lines. Consistent with this, the overexpression of the lowly expressed cadmium importers *SLC39A2* and *SLC39A8*, which did not score in the LOF screen, sensitized cells to cadmium, as shown in our cDNA-based overexpression screen. Based on our transcriptomic data, all the primary hits of the CRISPR screen (*ABCC1*, *SLC30A1*, and *SLC39A14*) are highly expressed in 1321N1 cells (data not shown). Furthermore, our CRISPR screens also showed a difference in the identification of transporters across the different heavy metals. Although we successfully identified a known importer of cadmium (SLC39A14), we identified an exporter (ABCC1) but no importer of arsenic and mercury. This observation raises the possibility that importers of arsenic and mercury comprise highly redundant, low-affinity membrane transporters, which might not score in a LOF screen. Furthermore, this discrepancy also implies a potential difference in mechanisms employed by cells to mitigate the toxicity of distinct metals. Specifically, cells mitigate the reactivity of cadmium through sequestration in chelating proteins, such as metallothioneins (Klaassen et al, 2009). However, cells employ efflux transporters to extrude arsenic and mercury (Vernhet et al, 2000; Garbinski et al, 2019), possibly because of the lower affinity of these metals for metallothioneins. This is also supported by the observation that cadmium treatment led to depletion of sgRNAs targeting metallothioneins and the upstream transcription factor *MTF1*. On the contrary, arsenic- and mercury-treated cells strongly depleted mutants lacking the glutathione reductase *GSR*, which is involved in the regeneration of glutathione and therefore required for the extrusion of glutathione–metal conjugates by ABCC1 (Leslie et al, 2004). In addition, both metals stimulated the expression of transporters required for the biogenesis of glutathione, emphasizing the overall importance of glutathione in mitigating the toxicity of arsenic and mercury (Leslie et al, 2004; Tokumoto et al, 2018).

The intertwined metabolism of zinc and cadmium has been widely studied in the last decades (Brzóska &Moniuszko-Jakoniuk, 2001). Because of their similar chemical properties, cadmium can largely interfere with the biological processes of zinc (Brzóska &Moniuszko-Jakoniuk, 2001). This disruption leads to dysregulation of cellular zinc homeostasis, contributing to several pathologies (Gasser et al, 2022; Arruebarrena et al, 2023). Conversely, numerous studies have shown that zinc counteracts cadmium-induced toxicity (Kaji et al, 1992; Pan et al, 2017). Here, by the unbiased interrogation of all membrane transporters, we showed that the strongest protection against cadmium toxicity is conferred by the KO of *SLC30A1*, through the modulation of intracellular zinc levels. Several studies have reported that metallothioneins serve as the first line of defense against cadmium toxicity, as also evidenced by observations in mice overexpressing or lacking metallothioneins (Liu et al, 1995; Park et al, 2001). Consistent with previous findings, we also observed that the induction of metallothioneins via pre-treatment with zinc, mimicking zinc accumulation in *SLC30A1*-KO cells, largely rescued cultured human cells from cadmium toxicity (Kennette et al, 2005). That metallothioneins are the main factor contributing to cadmium protection in *SLC30A1*-KO cells is also supported by the observation that inactivation of *SLC30A1* conferred a weaker protective effect against mercury toxicity, because metallothioneins have a weaker affinity to mercury, potentially due to prior conjugation of mercury to glutathione (Tokumoto et al, 2018). This may also be reflected by the weaker induction of metallothioneins in WT cells by mercury compared with cadmium, as well as the fact the cadmium but not mercury treatment led to depletion of sgRNAs targeting metallothioneins in the CRISPR screen. Moreover, zinc is a well-established antioxidant and it was previously shown that zinc pre-treatment suppresses cadmium-induced oxidative stress (Souza et al, 2004). The importance of zinc in counteracting cadmium toxicity is further highlighted by our cDNA-based overexpression screen, where we observed that the differential expression of several zinc transporters significantly determined the sensitivity to cadmium toxicity.

As already mentioned, an inherent limitation of genetic LOF screens is the genetic redundancy, which is especially pronounced among membrane transporters. However, given the pleiotropic effects of heavy metal toxicity, genetic screens represent an elegant approach to identify molecular targets and modulators of heavy metal toxicity in human cell lines (Peng et al, 2021; Tsvetkov et al, 2022). Recently, a genome-wide CRISPR/Cas9 screen in the human chronic myeloid leukemia cell line K562 identified *ABCC1*, *SLC30A1*, and *AQP3* as important regulators of arsenic toxicity (Sobh et al, 2019). Future studies should consider applying gain-of-function or combinatorial LOF genetic approaches to circumvent the redundancy (Girardi et al, 2020a; Rebsamen et al, 2022; Chidley et al, 2024). Moreover, additional focus should be set on ion channels, as several studies have highlighted their intricate interplay with heavy metals (Hinkle et al, 1987; Prabhu & Salama, 1990; Vijverberg et al, 1994).

Despite these limitations, our findings demonstrate that the genetic inactivation or overexpression of individual transporters can effectively alter the sensitivity of human cell lines to non-essential metals. This modulation occurs not only through direct changes in cellular metal accumulation but also through the acquisition of protective metabolites, such as zinc. Ultimately, our results underscore the potential of transporters as possible targets for pharmacological modulation of the chemical exposome, particularly given the significant impact of heavy metal toxicity on public health (Lefèvre-Arbogast et al, 2024; Sies et al, 2024).

## Materials and Methods

### Cell lines and reagents

HCT116, U937, hTERT-RPE1, and HEK293T cell lines were purchased from the ATCC, the 1321N1 cell line was obtained from Sigma-Aldrich, and the Huh7 cell line was acquired from the JCRB Cell Bank. U937 and HCT116 cells were maintained in RPMI 1640. HEK293T, Huh7, and 1321N1 cells were maintained in DMEM, and hTERT-RPE1 cells were cultured in DMEM:F12 containing 0.01 mg/ml hygromycin B (InvivoGen). All media (Sigma-Aldrich) were supplemented with 10% FBS (S1810; Biowest) and antibiotics (100 U/ml penicillin and 0.1 mg/ml streptomycin, P4333; Gibco), and cell lines were grown at 37°C in 5% CO_2_. Cell lines were checked for mycoplasma by PCR. For treatments with heavy metals, the heavy metal salts were dissolved in water and sterile-filtered, and cells were cultured in corresponding complete medium containing cadmium(II) chloride (202908; Sigma-Aldrich), mercury(II) chloride (215465; Sigma-Aldrich), sodium meta-arsenite (S7400; Sigma-Aldrich), manganese(II) chloride tetrahydrate (M3634; Sigma-Aldrich), or zinc chloride (96468; Sigma-Aldrich).

### Cell viability assay

For viability assays, cells were seeded in black 96-well plates (PhenoPlate, 6055302; PerkinElmer) and treated the next day with 60 μl heavy metal–containing complete medium. cDNA-overexpressing cell lines were seeded in medium containing 1 μg/ml doxycycline, which was maintained in the medium for the duration of the heavy metal treatment. If not otherwise stated, viability was measured after 48 h of treatment using the CellTiter-Glo assay (Promega) with a plate reader (SpectraMax i3x; Molecular Probes). Data were normalized to non-treated samples (100% viability), and four-parameter sigmoidal fitting curves were fitted using GraphPad Prism (v10.1.1). Each assay was conducted in a minimum of two technical replicates, and the results are expressed as the mean ± SD of at least three biological replicates.

### Quantification of cellular metal levels

Labile cellular zinc levels were determined using the fluorescent zinc sensor Zinpyr-1 (ab145349; Abcam). Cells were seeded in black 96-well plates (PhenoPlate, 6055302; PerkinElmer) pre-coated with poly-L-lysine hydrobromide (P6286; Sigma-Aldrich). The next day, the cells were washed with phenol red–free DMEM (A1443001; Gibco) without serum or antibiotics, supplemented with 25 mM glucose. The cells were then incubated with 10 μM Zinpyr-1 in the same medium for 30 min at 37°C in 5% CO_2_. After incubation, the cells were washed with phenol red–free DMEM and imaged using the Opera Phenix high-content screening system (PerkinElmer). The fluorescence intensity per cell was quantified using ImageJ (v1.0) by manually selecting individual cells.

Total cellular zinc and cadmium levels were determined through ICP-MS. The cells were seeded in 15-cm dishes and treated the next day with 1 μM cadmium chloride for 16 h. The cells were then harvested by trypsinization, washed three times with cold PBS buffer, and snap-frozen in liquid nitrogen. The cell pellet weight for subsequent data normalization was measured before freezing. Each sample (∼50–100 mg) was digested using 500 μl of nitric acid and 30 μl of H₂O₂. The microwave-assisted acid digestion was performed using a MW7000 system from Anton Paar. Elemental analysis was conducted using an iCAP quadrupole ICP-MS (Thermo Fisher Scientific). The system operated in kinetic energy discrimination mode with the reaction/collision cell filled with helium (4.1 ml/min). Cadmium was detected at m/z 111 and zinc at m/z 66. Multi-element standards were used for calibration to ensure accurate quantification.

### Membrane transporter–focused CRISPR/Cas9-KO screens

The transporter-focused library was designed with six sgRNAs per gene, specifically targeting 452 SLCs, 48 ABC transporter, 14 AQPs, and 40 P-type ATPase genes, along with 79 pseudogenes (comprising 76 SLCs, 2 ABC transporter, and 1 P-type ATPase). In addition, the library included control sgRNAs for 20 known essential genes, 20 olfactory receptors (as non-essential controls), and 60 random, non-targeting gRNAs (Table S1). Furthermore, we included sgRNAs targeting 26 genes involved in the regulation of heavy metal toxicity as controls for the metal treatment (Table S1). The design process involved using the VBC score (Michlits et al, 2020) or CRISPick (Doench et al, 2016; Sanson et al, 2018) tool for coding genes and pseudogenes, respectively. Typically, the selection criteria involved choosing the six top-ranked gRNAs while avoiding cut sites near the transcriptional start site or close to the 3′ end of the ORF. DNA oligonucleotides containing the sgRNA sequences were synthesized by Twist Bioscience and Integrated DNA Technologies. After synthesis, the oligos were amplified by PCR to generate double-stranded oligos. Subsequently, they were inserted between the two BsmBI restriction sites of a modified version of lentiCRISPRv2 (#52961; Addgene), which contains a stabilizing modification of the tracrRNA (Chen et al, 2013), the puromycin resistance is replaced with the blasticidin resistance, and the AAA trinucleotide located immediately upstream of the first BsmBI site is mutated to TGT. The transporter-focused library (without the sgRNAs targeting the heavy metal control genes) is available at Addgene (#213695). Lentiviral particles containing the CRISPR/Cas9-KO library were generated from HEK293T cells by transient transfection of 8.7 μg psPAX2 (#12260; Addgene), 5.8 μg pMD2.G (#12259; Addgene), and 11.7 μg sgRNA library using 145 μg PEI (Sigma-Aldrich) in serum-free DMEM. 16 h post-transfection, the medium was exchanged with DMEM complete medium, and 48 h after the start of the transfection, the viral supernatant was harvested, filtered (0.45 μm), and stored at −80°C. The 1321N1 cells were infected in sets of three replicates (for the genetic screens with arsenic and mercury) or four replicates (for the genetic screen with cadmium) using the viral supernatant (supplemented with 8 μg/ml protamine sulfate) at an MOI of ∼0.3 and an average 1000-fold library coverage. The infected cells were then selected for 4 d with 10 μg/ml blasticidin in DMEM, followed by recovery in blasticidin-free DMEM for 3 d. Subsequently, cells (4 × 10^6^ per condition) were exposed to toxic concentrations of arsenic (3.8 μM for 6 d), mercury (25 μM for 2 d, followed by 12.5 μM for 4 d), or cadmium (3.5 μM for 2 d, followed by 1 μM for 4 d), or left untreated. Untreated cells were passaged every 3 d. After 6 d of treatment, cells were harvested, washed with PBS, snap-frozen with liquid nitrogen, and stored at −80°C. Genomic DNA was isolated using the DNeasy kit (QIAGEN); the sgRNA sequences were amplified using pooled staggered primers, and the purified PCR products were used in a second PCR with Illumina indexing primers to generate Illumina libraries. The barcoded libraries were multiplexed and then sequenced on NovaSeq (Illumina) at the Biomedical Sequencing Facility (BSF at CeMM; https://www.biomedical-sequencing.org).

### cDNA-based overexpression screen

The SLC-focused cDNA overexpression library was generated previously (Wolf et al, manuscript submitted) by amplifying the codon-optimized cDNAs of 452 SLCs, the ABC transporters ABCC1, ABCB1, and ABCF2, eGFP, and a constitutively active MLKL variant (MLKLS358D) from pDONR221 plasmids (www.addgene.org/RESOLUTE_Consortium) with primers containing 10-bp barcodes (Table S3). The PCR products were purified with AmpliClean magnetic beads (NimaGen) and cloned into a pLIX_401 vector (#41393; Addgene) using a GenBuilder cloning kit (GenScript). The cDNA library is available at Addgene (#213694). Lentiviral particles containing the cDNA library were generated from HEK293T cells by transient transfection of 17.5 μg psPAX2 (#12260; Addgene), 12.5 μg pMD2.G (#12259; Addgene), and 25 μg cDNA library using 220 μg PEI (Sigma-Aldrich) in serum-free DMEM. For the cadmium survival screen, the 1321N1 cells were infected in sets of four replicates using the viral supernatant (supplemented with 8 μg/ml protamine sulfate) at an MOI of ∼0.3 and an average 3,300-fold coverage for each barcoded cDNA. Infected cells were selected for 4 d with 0.7 μg/ml puromycin in DMEM, followed by recovery in puromycin-free medium for 3 d. For each condition, ∼1.1 × 10^6^ cells were seeded in 1 μg/ml doxycycline-containing medium, and the next day exposed to toxic concentrations of cadmium or left untreated, both in the presence of doxycycline. Untreated cells were passaged every 3 d. After 5 d of treatment, cells were harvested, washed with PBS, snap-frozen with liquid nitrogen, and stored at −80°C. Genomic DNA was isolated using the DNeasy kit (QIAGEN); the cDNA barcode sequences were amplified using pooled staggered primers, and the purified PCR products were used in a second PCR with Illumina indexing primers to generate Illumina libraries. Subsequently, the libraries were multiplexed and then sequenced on NovaSeq (Illumina) at the BSF (at CeMM; https://www.biomedical-sequencing.org).

### Analysis of the genetic screens

The analysis of the cDNA overexpression and the CRISPR-KO screens was performed with tools from the Galaxy platform (Galaxy Community, 2022). The demultiplexed NGS reads were trimmed with cutadapt (v4.6) (Martin, 2011) to retrieve the cDNA barcode or the sgRNA sequences, and the reads were mapped and counted using MAGeCK count (v0.5.9.2.4) (Li et al, 2014) and normalized on total read counts. For the CRISPR screens, the differential enrichment of sgRNA in treated compared with non-treated samples was calculated with a MAGeCK test (v0.5.9.2.1) by applying normalizations on total read counts and FDR-based *P*-value adjustment. The log fold change was determined by calculating the median of the sgRNA enrichment for each gene. For the overexpression screen, the enrichment of each cDNA was determined using the DESeq2 tool (v2.11.40.8) with default parameters (Love et al, 2014). Data visualizations were carried out using R-project (v4.2.2; R Foundation for Statistical Computing; https://www.R-project.org/) with RStudio (v2023.03.0+386; Integrated Development for R; http://www.rstudio.com/), dplyr (v1.1.1), tidyverse (v2.0.0), ggplot2 (v3.4.2), and ggrepel (v0.9.2).

### Plasmids and cell line generation

For the generation of KO cell lines, we applied the modified lentiCRISPRv2 vector described above. For each gene of interest, two sgRNAs from our transporter-focused KO library were selected, and the corresponding forward and reverse oligos, flanked by BsmBI-cutting sites, were annealed and cloned into the modified lentiCRISPRv2 vector via Golden Gate assembly (Table 2). sgOR1A1 targeting the olfactory receptor *OR1A1* was used as a cutting, negative control sgRNA. For the generation of doxycycline-inducible overexpression cell lines, we used pLIX plasmids (#194066; Addgene) containing the cDNAs of *SLC30A1* and *SLC39A14* tagged with a C-terminal Strep-HA tag, which were obtained from the RESOLUTE consortium.

**Table 2.** sgRNA sequences for the generation of KO cell lines.

sgSLC39A14_1	GTGCTGGGTGACATTACCC
sgSLC39A14_2	GAGTGCCGATATCAGAGTAG
sgSLC30A1_1	GATCCGAGCCGAGGTAATGG
sgSLC30A1_2	GCCAGCAGGATGGCGAAACAG
sgSLC12A9_1	GATTCCGATCAACACCAGTG
sgSLC12A9_2	GGTGAAGTGGCCAAACCG
sgABCC1_1	GAACAAGGCCAAAGACACGA
sgABCC1_2	GTGTACTGGGACTACATGA
sgOR1A1	GTTTCCAATCAATGTGATG

The 1321N1-KO and cDNA overexpression cell lines were generated through lentiviral transduction. In short, HEK293T cells were transfected with 4.2 μg psPAX2 (#12260; Addgene), 3 μg pMD2.G (#12259; Addgene), and 6 μg sgRNA or cDNA plasmid using 60 μg PEI (Sigma-Aldrich), and after 16 h of transfection, the medium was replaced with DMEM complete medium. The viral supernatant was harvested 48 h after the start of the transfection, filtered (0.45 μm), and stored at −80°C. Subsequently, cells were infected with the virus in a ratio 1:3 (vol/vol) supplemented with 8 μg/ml protamine sulfate. At 24 h post-infection, the medium was changed, and 48 h post-infection, cells underwent selection with the corresponding antibiotics. For each round of KO cell line generation, a corresponding control cell line bearing sgOR1A1 was concurrently generated.

The intron-tagged mScarlet-Metallothionein 1E (*mScarlet-MT1E*) 1321N1 cell line was generated as described previously (Reicher et al, 2020). In short, sgRNA targeting the first intron of *MT1E* (sgMT1E_Intron GAAAGCATCTAACGAAGTAC) was designed with Benchling, and corresponding forward and reverse oligos, flanked by BbsI-cutting sites, were annealed and cloned into pX330-Cas9 (#42230; Addgene) via Golden Gate assembly. 1321N1 parental cells were seeded in a six-well plate and co-transfected with 750 ng of pX330-Cas9 containing the MT1E intron–targeting sgRNA, 750 ng of pX330-Cas9 containing the donor-targeting sgRNA (#159741; Addgene), and 60 ng of the mScarlet donor minicircle (gifted from S Kubicek Lab) using PEI. After 16 h, the medium was replaced with DMEM supplemented with 10% FBS, and cells were kept in culture and passaged every 2–3 d. After 6 d, cells were sorted on a CytoFLEX SRT cell sorter, collecting mScarlet-positive cells, which were then expanded for further experiments.

### Confocal fluorescence microscopy

The mScarlet-MT1E 1321N1 cells were seeded in black 96-well plates (PhenoPlate, 6055302; PerkinElmer), and, if not otherwise stated, after 24 h of heavy metal treatment, the confluent cells were imaged with the Opera Phenix high-content screening system (PerkinElmer), acquiring 15 images per well. Subsequently, the fluorescence intensity was determined with Harmony High-Content Imaging and Analysis Software (PerkinElmer, v5.1), by averaging the mean fluorescence intensity of 15 images per well. Each assay was conducted in a minimum of two technical replicates, and the results are expressed as the mean ± SD of three biological replicates normalized on non-treated conditions.

### Transcriptomic analysis of cells treated with arsenic, cadmium, and mercury

1321N1 cells were seeded in six-well plates in DMEM complete medium. After 24 h, the medium was replaced with DMEM containing heavy metals at a concentration leading to 20% cell death, as determined for 48 h treatment (2.3 μM cadmium, 2.55 μM arsenic, and 20 μM mercury). All treatments were performed in triplicates. Cells were washed with cold PBS and lysed directly in the plate using the lysis buffer provided by the RNeasy kit (QIAGEN) at time points 1.5, 3, 6, 12, and 24 h. Non-treated conditions were included for each time point. Total RNA was isolated using the QIAGEN RNeasy kit including the on-column DNase I digestion. Subsequently, RNA-sequencing libraries were generated following the QuantSeq 3′ mRNA-Seq FWD library preparation protocol (Lexogen). Library concentrations were quantified with the Qubit 2.0 Fluorometric Quantitation system (Life Technologies), and the size distribution was assessed using the 2100 Bioanalyzer instrument (Agilent). Subsequently, samples were diluted and pooled into NGS libraries in equimolar amounts and were sequenced on a NovaSeq 6000 instrument (Illumina) at the BSF (at CeMM; https://www.biomedical-sequencing.org) following a 100-base pair single-end recipe. NGS reads were mapped to the Genome Reference Consortium GRCh38 assembly via “Spliced Transcripts Alignment to a Reference” (STAR, v2.7.9a) (Dobin et al, 2013) using the “basic” GENCODE transcript annotation from version 43 (February 2023) (Frankish et al, 2021) as a reference transcriptome. STAR was run with options recommended by the ENCODE project. NGS read alignments overlapping GENCODE exon features were counted with the Bioconductor (v3.16) GenomicAlignments (v1.34.0) package via the summarizeOverlaps function in Union mode, ignoring secondary alignments and alignments not passing vendor quality filtering, and alignments were counted strand-specifically in feature (i.e., gene, transcript, and exon) orientation. Exon-level counts were aggregated to gene-level counts, and the Bioconductor DESeq2 (v1.38.0) package (Love et al, 2014) was used to test for differential expression by comparing the treated samples with its corresponding non-treated samples at each time point based on a model using the negative binomial distribution. The log_2_ fold change values were shrunk with the CRAN ashr (v2.2.-54) package (Stephens, 2017), whereas two-tailed *P*-values obtained from Wald’s testing were adjusted with the Bioconductor Independent Hypothesis Weighting (v1.16.0) package (Ignatiadis et al, 2016). The resulting gene lists were annotated and filtered for significantly differentially up- and down-regulated genes.

### Gene set enrichment and transcription factor target gene enrichment analyses

GSEA (Subramanian et al, 2005) was conducted on the transcriptomic dataset of heavy metal–treated 1321N1 cells using the clusterProfiler package (v4.6.2) (Yu et al, 2012) in RStudio (v2023.03.0+386; Integrated Development for R; http://www.rstudio.com/) with R-project (v4.2.2; R Foundation for Statistical Computing; https://www.R-project.org/). In short, the differentially expressed gene dataset was filtered for the background gene set, defined as genes with FPKM > 1 in non-treated cells, and was then sorted by decreasing LFC. GSEA was performed using the Kyoto Encyclopedia of Genes and Genomes (Ogata et al, 1999), with a p-value cutoff of 0.05, applying the Benjamini–Hochberg procedure. The time point 1.5 h for arsenic- and cadmium-treated cells was excluded from the analysis because of the absence of significant DEGs with LFC > 1 or < −1. The enriched terms that rank highest were chosen according to the normalized enrichment score (NES) and displayed in descending order based on the sum of the NES for each term, whereas disease-related terms were omitted (Fig S1B). For the analysis of enriched transcription factor target genes, we filtered for significant DEGs with LFC > 1 or < −1, and calculated the relative enrichment of target genes based on the TRRUST dataset (v2) (Han et al, 2018), by normalizing the number of overlapping genes to the number of total targets of each transcription factor.

### siRNA-mediated knockdown of MT1E

The knockdown of MT1E was achieved using two different Dicer-substrate siRNAs (Integrated DNA Technologies): siRNA#1 CUGGAUUUUUUUAAAAAUACAACA and siRNA#2 GAUUUUUUUAAAAAUACAACACUGA. For the transfection, 30 pmol siRNA and 6 μl Lipofectamine RNAiMAX (#13778-150; Invitrogen) were pre-mixed in serum-free DMEM, and the transfection mix was added to the cells in a six-well plate format to a final volume of 3 ml DMEM full medium. After 24 h of transfection, the cells were seeded in black 96-well plates (PhenoPlate, 6055302; PerkinElmer) and treated with cadmium the next day for 48 h, followed by viability measurements. A non-targeting siRNA (IDT #51-01-14-04) was used as a negative control siRNA. The knockdown efficiency at the start and at the end of the heavy metal treatment was determined by qRT-PCR.

### qRT-PCR

The total RNA of cells transfected with siRNA targeting *MT1E* or the negative control siRNA was isolated using the RNeasy kit (QIAGEN) at 1 and 3 d post-transfection. The RNase inhibitor RiboLock (EO0381; Thermo Fisher Scientific) was included in all the following steps. Genomic DNA was removed via DNase I digestion (EN0521; Thermo Fisher Scientific) at 37°C for 30 min, followed by DNase inactivation with 4.5 mM EDTA at 65°C for 10 min. Then, 500 ng RNA was reverse-transcribed using RevertAid Reverse Transcriptase (EP0441; Thermo Fisher Scientific) with an equimolar mixture of oligo dT (12-, 15-, and 18-mer) and random hexamer primers (SO142; Thermo Fisher Scientific). Subsequently, the quantitative PCR was performed on the Bio-Rad CFX Opus 384 Dx PCR machine using Luna Universal qRT-PCR Master Mix (M3003S; NEB). The results were quantified with the 2^ΔΔCt^ method with *HPRT1* serving as the reference gene. The primers used were as follows: *MT1E* fwd: TCCTGCAAGAAGAGCTGCTG, rev: AAAAAGAAATGCAGCAAATGGC; *HPRT1* fwd: AGACTTTGCTTTCCTTGGTCAG, rev: CCAACAAAGTCTGGCTTATATCC.

## Supplementary Material

Reviewer comments

## Data Availability

The transcriptomic data from this publication have been deposited to the GEO database (https://www.ncbi.nlm.nih.gov/geo/) and assigned the identifier GSE281225.
